# HS–SPME–GC–MS and Electronic Nose Reveal Differences in the Volatile Profiles of *Hedychium* Flowers

**DOI:** 10.3390/molecules26175425

**Published:** 2021-09-06

**Authors:** Yiwei Zhou, Farhat Abbas, Zhidong Wang, Yunyi Yu, Yuechong Yue, Xinyue Li, Rangcai Yu, Yanping Fan

**Affiliations:** 1 The Research Center for Ornamental Plants, College of Forestry and Landscape Architecture, South China Agricultural University, Guangzhou 510642, China; zhouyiwei6333@163.com (Y.Z.); farhatmerani@yahoo.com (F.A.); zd.wang@founder.com (Z.W.); hyphen950@163.com (Y.Y.); yyc19871017@163.com (Y.Y.); lixinyue1203@126.com (X.L.); 2 College of Life Sciences, South China Agricultural University, Guangzhou 510642, China; rcyu@scau.edu.cn; 3 Guangdong Key Laboratory for Innovative Development and Utilization of Forest Plant Germplasm, South China Agricultural University, Guangzhou 510642, China

**Keywords:** *Hedychium*, floral volatile compounds, GC–MS, Electronic nose

## Abstract

Floral fragrance is one of the most important characteristics of ornamental plants and plays a pivotal role in plant lifespan such as pollinator attraction, pest repelling, and protection against abiotic and biotic stresses. However, the precise determination of floral fragrance is limited. In the present study, the floral volatile compounds of six *Hedychium* accessions exhibiting from faint to highly fragrant were comparatively analyzed via gas chromatography–mass spectrometry (GC–MS) and Electronic nose (E-nose). A total of 42 volatile compounds were identified through GC–MS analysis, including monoterpenoids (18 compounds), sesquiterpenoids (12), benzenoids/phenylpropanoids (8), fatty acid derivatives (2), and others (2). In *Hedychium coronarium* ‘ZS’, *H*. *forrestii* ‘Gaoling’, *H*. ‘Jin’, *H.* ‘Caixia’, and *H.* ‘Zhaoxia’, monoterpenoids were abundant, while sesquiterpenoids were found in large quantities in *H*. *coccineum* ‘KMH’. Hierarchical clustering analysis (HCA) divided the 42 volatile compounds into four different groups (I, II, III, IV), and Spearman correlation analysis showed these compounds to have different degrees of correlation. The E-nose was able to group the different accessions in the principal component analysis (PCA) corresponding to scent intensity. Furthermore, the pattern-recognition findings confirmed that the E-nose data validated the GC–MS results. The partial least squares (PLS) analysis between floral volatile compounds and sensors suggested that specific sensors were highly sensitive to terpenoids. In short, the E-nose is proficient in discriminating *Hedychium* accessions of different volatile profiles in both quantitative and qualitative aspects, offering an accurate and rapid reference technique for future applications.

## 1. Introduction

*Hedychium* belongs to the family Zingiberaceae and is an economically important crop grown extensively for its ornamental and medicinal properties. The flowers of *Hedychium* are widely used in perfumed and cosmetic industries, while rhizome is used for medicinal purposes [1,2,3]. There are more than 80 *Hedychium* species that have been reported; however, new species are still emerging [4,5,6]. *Hedychium* species are diverse in color with numerous shapes. With respect to their floral scent, *Hedychium* species vary from scentless to rich in fragrance with high ornamental values [6,7,8]. The flowers of *Hedychium* are rich in an aroma that is mainly composed of a mixture of monoterpenoids, sesquiterpenoids, and benzenoids [9,10,11,12]. Recently, the main focus has been on the physio-biochemical properties of *Hedychium*; however, there are few reports on the qualitative and quantitative analysis of the floral volatile profiles of *Hedychium*.

Flower fragrance is one of the key characteristics of flowering plants that play a crucial role in plant life. It functions in the reproductive processes of numerous plants, repels pests, and protects against pathogens as well as biotic and abiotic stresses [13,14,15]. Floral scent is also a key form of communication between plants and pollinators [16,17,18]. Furthermore, volatile compounds are extensively used in the cosmetic, food, and pharmaceutical industries and biofuel products [19,20,21]. Fragrance not only increases the aesthetic values of ornamental plants, but the relaxing and appealing aroma of flowers can also be used in psychological and physiological treatments [22].

The quantitative and qualitative analysis of floral fragrance is crucial to predicting and better understanding flower visitors’ behavior. Recently, various chromatographic methods such as gas chromatography–mass spectrometry (GC–MS) and headspace analysis have been widely used to identify and quantify the aromatic components of flowering plants. To assess the volatile profile of ornamental plants, the aforementioned techniques have been used in *Silene latifolia* [23], *Lantana canescens* Kunth [24], *Rosa damascene* [25], *H. coronarium* [26,27], *Luculia pinceana* [28], *Lilium* ‘Siberia’ [29,30,31], and *Osmanthus fragrans* [32]. Previously, the headspace solid-phase microextraction (HS–SPME) GC–MS technique was used to determine the volatile components of *Hedychium* [33,34,35]; however, it retains shortcomings such as prolonged analysis time, high running cost, and complex technology as compared to E-nose. Although the fully automated HS–SPME–GC–MS machines are available, the price of the machine and running cost of the sample is high compared to E-nose. These techniques usually fail to give a global fingerprint of the aroma sample, as the detection of compounds is dependent on the sample pretreatment method, which is why careful attention needs to be paid to it. The Electronic nose (E-nose) is another method that has been used extensively in medical diagnosis [36], product quality testing [37], and monitoring of the environment [38]. E-nose machines perform complex pattern recognition similar to the human olfactory system. Furthermore, the E-nose system can identify the presence of volatile organic compounds of various molecular structures with odor reliability and high accuracy, no matter the intensity of the odor. It can also perform a quantitative and qualitative analysis of odor samples [39]. However, the E-nose system also has limitations regarding the identification of volatile compounds. Recently, the E-nose system has been used in various studies to determine the floral fragrance among different species and to distinguish between flowering stages and different floral organs [40,41,42]. However, there is no report regarding the use of an E-nose system to evaluate the floral fragrance in *Hedychium*.

In the current study, HS–SPME–GC–MS was used to determine and analyze the volatile profiles of *Hedychium* flowers. At the same time, the ability of the E-nose to distinguish different volatile profile was evaluated. Finally, the relationship between volatile compounds and the E-nose sensors was explored through various analyses, which will assist in the rapid detection of scent type classification and thus improve the scented flower breeding system.

## 2. Results

### 2.1. Chemical Composition of Floral Volatiles Analyzed via HS–SPME–GC–MS

**Figure 1:**
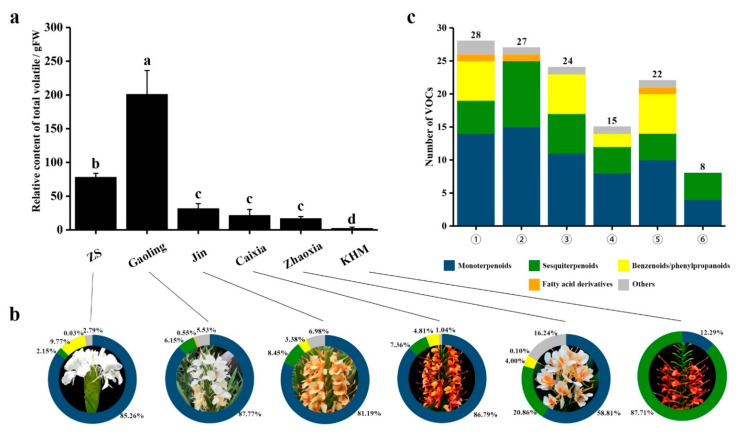
Content, classification, and difference in VOCs identified by HS–SPME–GC–MS in six *Hedychium* accessions. (**a**) The total floral volatile contents of six *Hedychium* accessions. a, b, c, and d refer to the significant difference (*p* < 0.05). (**b**) The proportion of different VOCs in six *Hedychium* accessions. ①: ‘ZS’; ②: ‘Gaoling’; ③: ‘Jin’; ④: ‘Caixia’; ⑤: ‘Zhaoxia’; ⑥: ‘KMH’. (**c**) The number of volatile organic compounds present in the floral volatile profiles of six *Hedychium* accessions. Different letters indicate significant differences among means according to ANOVA analysis (*p* < 0.05). Relative contents (%) = (area under peak/total peak area) × 100; all data are presented as mean ± SD (n = 3).

To determine the floral volatile compounds of *Hedychium* accessions, their volatile compounds were subjected to HS–SPME–GC–MS, and the identified volatile organic compounds (VOCs) were compared (Supplementary Figure S1). The identified VOCs and their corresponding amounts are summarized in Table 1. A total of 42 VOCs were identified in the floral profile of six *Hedychium* accessions, including 18 monoterpenoids, 12 sesquiterpenoids, 8 phenylpropanoids, 2 fatty acid derivatives, and 2 other categories (Table 1). A significant difference in the relative contents and types of VOCs in the flowers was observed in *Hedychium* accessions. The results showed that the amount of VOCs was highest in *H. forrestii* ‘Gaoling’, followed by *H. coronarium* ‘ZS’, *H*. ‘Jin’, *H*. ‘Caixia’, *H*. ‘Zhaoxia’, and *H. coccineum* ‘KMH’ (Figure 1a). Furthermore, monoterpenoids were the primary VOCs of ‘ZS’, ‘Gaoling’, ‘Jin’, ‘Caixia’, and ‘Zhaoxia’, while sesquiterpenoids were foremost in ‘KMH’ (Figure 1b). The main volatile components in ‘ZS’ were monoterpenoids (85.26%) and benzenoids/phenylpropanoids (9.77%). In ‘Gaoling’, monoterpenoids (87.77%) along with sesquiterpenoids (6.15%) were the primary components, while monoterpenoids (81.19%) and sesquiterpenoids (8.45%) were the main components in ‘Jin’. Similarly, the total volatile components of ‘KMH’ were sesquiterpenoids (87.71%) and monoterpenoids (12.29%). Moreover, benzenoid/phenylpropanoid contents were missing from the volatile profiles of ‘Gaoling’ and ‘KMH’, while fatty acids were missing from ‘Jin’, ‘Caixia’, and ‘KMH’.

Among the six *Hedychium* accessions, ‘ZS’ had the largest number of volatile compounds with 28 types, followed by ‘Gaoling’ (27 types), ‘Jin’ (24 types), ‘Zhaoxia’ (22 types), ‘Caixia’ (15 types), and ‘KMH’ (8 types) (Figure 1c; Supplementary Figure S2). There were six ((*Z*)-β-terpineol, calarene, 2-norpinene, β-himachalene, nerolidol, and isobornyl acetate) unique compounds in ‘Gaoling’. With respect to floral volatile composition, linalool and (*E*)-β-ocimene constituted 62.34%, 67.05%, 77.93%, and 48.19% of the total volatiles of ‘ZS’, ‘Jin’, ‘Caixia’, and ‘Zhaoxia’, respectively (Supplementary Table S1). Eucalyptol and (*E*)-β-ocimene contributed 68.41% to the total volatiles of ‘Gaoling’, while caryophyllene and β-farnesene constituted 76.75% of ‘KMH’. In short, terpenoids were the main volatile compounds in the volatile profiles of *Hedychium* accessions. Interestingly, three ((*E*)-β-ocimene, linalool, caryophyllene, (*E*)-β-farnesene, α-farnesene) compounds were shared among six *Hedychium* accessions (Table 1; Supplementary Figure S2).

### 2.2. Hierarchical Clustering Analysis (HCA) Based on GC–MS Data

**Figure 2:**
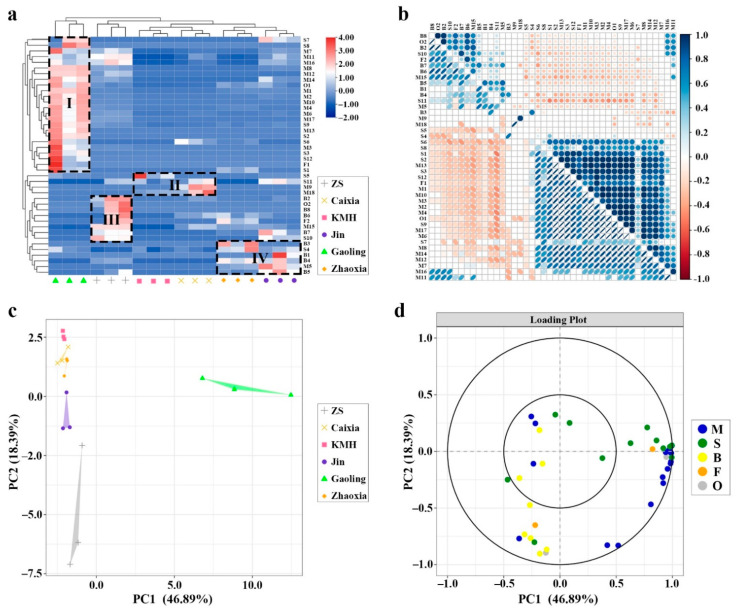
Multivariate analysis of floral volatile profiles of six *Hedychium* accessions assessed by HS–SPME–GC–MS. (**a**) Heat map of volatile compounds in six *Hedychium* accessions. (**b**) Correlation matrix of 42 VOCs (significant (*p* < 0.05) combinations are displayed). (**c**) The distribution of VOCs of six *Hedychium* accessions over the PCA score plot defined by the first two principal components. (**d**) The projection of the VOCs identified by HS–SPME–GC–MS analysis. Each compound is shown in a different color (monoterpenoids (M), sesquiterpenoids (S), benzenoids/phenylpropanoids (B), fatty acid derivatives (F), others (O)).

To show the differences in the VOCs among *Hedychium* accessions, HCA analysis was performed (Figure 2a). The red color indicates a higher than average content value, while the blue color in the plot indicates a lower content value. The data showed that the contents of the VOCs (42 compounds) from six *Hedychium* accessions could be divided into four clusters (I, II, III, and IV) (Figure 2a). Cluster I include 24 compounds with high contents in the medium scented ‘Gaoling’. Cluster II includes four compounds with high content in the faintly fragrant ‘KMH’ ((*E*)-β-farnesene) and the weakly fragrant ‘Caixia’ (limonene, dihydro-β-ionone, δ-cadinene). Cluster III includes eight compounds (linalool, α-amorphene, methyl benzoate, phenol, 2-methoxy-4-(1-propenyl), 1-butanol, 3-methyl, benzoate, benzyl benzoate, methyl jasmonate, and indole) with high content in the strongly fragrant ‘ZS’. Likewise, cluster IV includes six compounds (β-pinene, caryophyllene, anisole, phenylethyl alcohol, benzyl nitrile, and eugenol) with high content in the weak scented ‘Zhaoxia’ and ‘Jin’. Furthermore, Spearman correlation analysis showed that 42 VOCs had different degrees of correlation (Figure 2b).

### 2.3. Principal Component Analysis Based on GC–MS Data

To analyze the floral volatile compounds that play a crucial role in differentiating volatile profile, the 42 VOCs identified via HS–SPME–GC–MS were subjected to principal component analysis. The results showed that 65.28% of the total variability was accounted for by the first two principal components, and six accessions could be distinguished (Figure 2c). Moreover, terpenoids (monoterpenoids and sesquiterpenoids) contributed mainly to PC1, while benzenoids/phenylpropanoids contributed to PC2 (Figure 2d).

### 2.4. Discrimination of the Different Taxa Using the E-Nose

**Figure 3:**
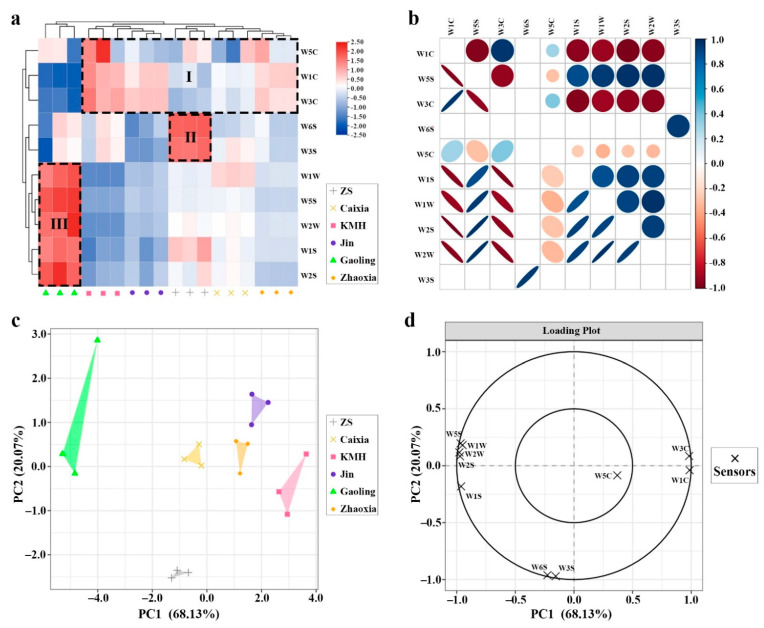
Multivariate analysis of floral volatile profile of six *Hedychium* accessions assessed by E-nose. (**a**) Heat map of response values of ten E-nose sensors in six *Hedychium* accessions. (**b**) Correlation matrix of ten E-nose sensors (significant (*p* < 0.05) combinations are displayed). (**c**) The volatile profile distribution of six *Hedychium* accessions over the PCA score plot defined by the first two principal components. (**d**) The projection of the scent identified by E-nose sensor analysis.

In the current study, the floral volatile profiles of six *Hedychium* accessions were measured via E-nose, and the mean sensor values are listed in Table 2. The results showed that sensors W1W, W2W, and W5S were highly responsive when compared to other sensors. Moreover, the response values of the aforementioned sensors were highest for ‘Gaoling’, followed by ‘Caixia’, ‘ZS’, ‘Zhaoxia’, ‘Jin’, and ‘KMH’ (Table 2). HCA analysis divided the ten sensors into three groups. Group I includes three sensors (W5C, W1C, and W3C) with high response values to ‘Jin’, ’Zhaoxia’, and ’KMH’, Group II includes two sensors (W6S and W3S) with higher responsive values to ‘ZS’, while Group III includes five sensors (W1S, W2S, W5S, W1W, and W2W) with high response value to ‘Gaoling’ (Figure 3a). Correlation analysis showed that sensor W1C was positively correlated with W3C and W5C, while W1C and W3C were negatively correlated with W1S, W2S, W1W, W2W, and W5S (Figure 3b; Table 2). In addition, there was a significant positive correlation among W1S, W2S, W5S, W1W, and W2W (Figure 3b). Principal component analysis showed that the first two principal components explained 88.20% of the variance, and six accessions with different floral volatile profiles could be distinguished (Figure 3c). Sensors W1C, W3C, W1S, W2S, W5S, W1W, and W2W contributed greatly to PC1, while sensors W3S and W6S mainly explained the difference in PC2 (Figure 3d). Meanwhile, W5C contributed less toward both PC1 and PC2.

### 2.5. Correlation between GC–MS and E-Nose Sensors

**Figure 4:**
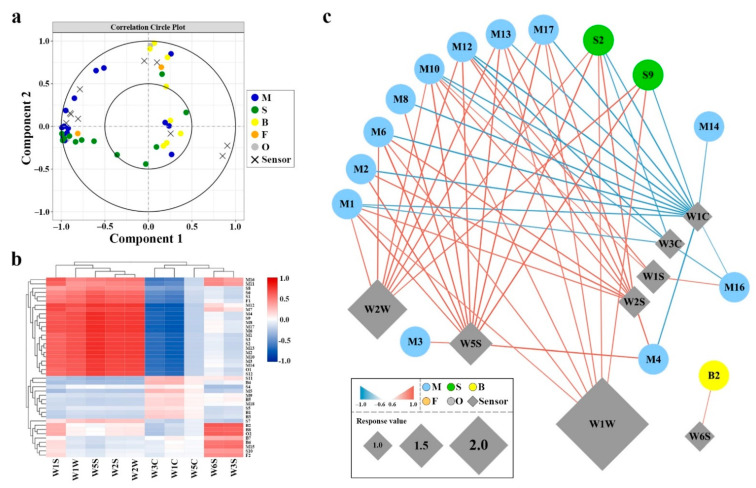
PLS regression of VOCs data obtained by HS–SPME–GC–MS and E-nose analysis. Plot (**a**) reports the loading plot of the PLS regression analysis assessed with the HS–SPME–GC–MS and E-nose data. Plot (**b**) shows the heatmap of the PLS correlation coefficients between VOCs and sensors. Plot (**c**) discloses the correlation analysis network based on significance (*p* < 0.05). PLS correlations between VOCs detected by HS–SPME–GC–MS and sensors by E-nose. The gradient color coding of the edges and the line thickness denote the level of correlation (0.6–1). Positive and negative correlations are shown by red and blue gradient colors.

To analyze the correlation between GC–MS and E-nose measurements, a PLS regression analysis was performed (Figure 4). The results revealed that the sensors showed different responses to different volatiles. Thirty-one volatile compounds and nine sensors are located in the right section of the plot, and they explained between 50 and 100% of the cross-validated variance, suggesting a significant positive correlation between these variables (*p* < 0.05) (Figure 4a). The heatmap of the PLS correlation coefficients showed that sensors W1S, W1W, W5S, W2W, W6S, and W3S were positively correlated with the VOCs, while sensors W3C and W1C had negative correlation coefficients (Figure 4b). Sensor W2W was positively correlated with 6 monoterpenoids and 2 sesquiterpenoids. Sensor W1W was positively correlated with 5 monoterpenoids and 2 sesquiterpenoids. The sensors W1S, W2S, and W5S were positively correlated with 3, 8, and 12 compounds, respectively (Figure 4c). Meanwhile, sensors W3C and W1C were negatively correlated with 5 and 13 compounds, respectively. Interestingly, sensor W6S was significant positively correlated with methyl benzoate.

## 3. Discussion

*Hedychium* is a perennial herb grown extensively as a garden plant as well as a cut flower in both tropical and subtropical regions. The flower blossoming results in the emission of abundant volatile compounds composed mainly of terpenes and benzenoids/phenylpropanoid substances [7,8,10,12,26]. Several accessions with high ornamental values are present in the market that range from scentless (*H*. *coccineum*) to highly fragrant (*H*. *coronarium*) [7,43]. The precise determination of volatile compounds and biosynthesis of floral volatile components will assist researchers in the *Hedychium* breeding program. In this study, Electronic nose technology was used for the first time to evaluate the volatile components of *Hedychium,* along with HS–SPME–GC–MS. Differences in the volatile profiles of six *Hedychium* accessions were evaluated, as volatile components are affected by their genetic contexts.

To measure the floral volatile contents and to analyze the differences among the floral volatile profiles of *Hedychium* accessions, the HS–SPME–GC–MS approach was used. The GC–MS data showed that the types and contents of VOCs in *Hedychium* accessions varied significantly (Table 1). Similarly, significant differences in volatile compounds were observed in *Lilium* and *Anthurium* [44,45]. A total of 42 volatile compounds were identified, and terpenoids were the main volatile contents in six *Hedychium* accessions, which is in agreement with previous studies [7,8,10]. Furthermore, linalool and (*E*)-β-ocimene constituted 62.34% of the total volatiles in ‘ZS’, which is in line with the previous findings that linalool and (*E*)-β-ocimene are the main floral volatiles of this species [43,46]. The HCA analysis data showed that 42 volatile compounds from *Hedychium* accessions could be categorized into four clusters, while Spearman analysis revealed that these VOCs had different degrees of correlation (Figure 2). Furthermore, PCA data showed that floral volatile compounds were distributed among all accessions and that six accessions could be distinguished. These data demonstrated that monoterpenoids and sesquiterpenoids had a significant influence on the floral characteristics of *Hedychium* accessions. Previous studies also showed that the expression of genes related to the biosynthesis of terpenoids in *Anthurium* [45], *Lilium* ‘Siberia’ [29,30,31], and *Freesia* [42] had a certain correlation with the volatilization of terpenoids. In the future, the relationship between genes related to the biosynthesis pathway of terpenoids and their volatile compounds will be worth exploring in *Hedychium*.

The analysis of floral volatiles via E-nose requires little time, and the system is easy to operate, while sensory analysis via expert panel is costly [47,48]. Hence, the E-nose system was selected as a fast and qualitative technique to study the differences in the floral volatile profile of *Hedychium*. Previous studies have shown that the E-nose system can accurately detect the VOCs of various molecular structures regardless of the odor intensity [49]. In this study, there was no linear relationship between the data measured by E-nose and the intensity of the aroma, but there was consistency between the data measured by E-nose and the total amount of volatile compounds determined by GC–MS. This might be due to the influence of the properties of different compounds and the olfactory threshold. Many studies have shown that various volatile compounds have different degrees of correlation with different E-nose sensors [50,51]. Herein, the differences among the volatile profiles of *Hedychium* accessions were observed. Through PLS regression analysis, we observed that W1W, W2W, and W5S sensors with high response values had different degrees of correlation with monoterpenoids and sesquiterpenoids, suggesting that E-nose is an effective method to evaluate floral volatile profiles of *Hedychium* (Figure 4). Furthermore, sensor W6S was responsive to benzenoids/phenylpropanoids. In *Actinidia*, E-nose sensors S7, S10, S8, S6, S9, and S2 were highly responsive to terpene compounds [52]. Similar findings were observed in *Prunus persica*, where the E-nose accurately distinguished peach samples of two different flesh colors [53]. In *Malus* taxa, the nitrogen-containing compounds, terpenes, and sulfur-containing compounds were highly correlated with sensors W5C, W1W, and W2S [54]. These findings suggested that E-nose is an effective and rapid technique for studying the floral volatile profile of *Hedychium* flowers.

The evaluation of flower aroma is very complex, and the detection of floral fragrance is one of the key limitations of flower fragrance breeding programs. Previously, researchers have relied mainly on the GC–MS system to identify the floral components in ornamental plants; however, there have been few reports on the use of E-nose [55] and PTR–MS [56] to identify the floral volatile profiles of ornamental plants. Each method has its advantages and disadvantages; however, the combination of GC–MS with E-nose or PTR–MS can efficiently discriminate the aroma components [57]. Recently, a combination of E-nose and GC–MS, PTR–MS, and GC–MS joint analysis was used in *Cucumis melo* L. [58], *Actinidia* [52], *Prunus* [53], *Vaccinium* spp. [59], and several other plants to identify the VOCs. However, there are few reports on the combined analysis of multi-methods in flower fragrance. In this study, the floral characteristics of different *Hedychium* accessions were analyzed efficiently by combined analysis using GC–MS and E-nose. Herein, we provided a reference for the establishment of the rapid detection of *Hedychium* floral volatile profiles. In the future, the combination of different detection methods can be applied to precisely identify and measure the floral VOCs of ornamental plants.

## 4. Materials and Methods

### 4.1. Plant Materials

The scent intensity of floral volatile compounds from *Hedychium* accessions was assessed by 25 trained assessors, as described previously [60]. Based on the evaluation data, *H. coronarium* ‘ZS’, *H. forrestii* ‘Gaoling’, *H.* ‘Jin’ (hybrid cultivar), *H.* ‘Caixia’ (hybrid cultivar), *H.* ‘Zhaoxia’ (hybrid cultivar), and *H. coccineum* ‘KMH’ were categorized as highly fragrant, medium, weak, weak, weak, and faintly scented, respectively (Supplementary Table S2). The plants were grown under natural light conditions at South China Agricultural University, Guangzhou, China (23.16° N, 113.36° E). The plants were grown in the controlled greenhouse under conditions: 26 ± 2 °C and 75–80% humidity.

### 4.2. Sample Preparation and HS-SPME-GC–MS Analysis

The samples for volatile determination were collected in the morning from September to October. The individual fresh flower (2 g) was placed in a 250 mL glass bottle supplemented with an internal standard. The flower was enclosed in a 250 milliliter (mL) glass bottle with the addition of 1.728 micrograms (μg) ethyl caprate, which served as an internal standard. After this, the bottle was immediately closed with aluminum foil. After 30 min, polydimethylsiloxane (PDMS, with 50/30 µm divinylbenzene/Carboxen, Supelco) fiber was injected into the bottle for 30 min to trap the volatiles, which was followed by insertion into a GC–MS (Agilent) or E-nose system for volatile analysis, as described previously [11,43]. The HS-SPME extraction was performed at 26 ± 2 °C. The GC–MS system with Agilent 7890A GC and Agilent 5975C MSD was provided with an Agilent DB-5MS capillary column (30 m × 0.25 mm), and helium gas was provided as a carrier. The flow of helium gas was kept constant at 1 mL/min. Initially, the GC injection port temperatures was maintained at 40 °C for 3 min, which was followed by an increase in temperature of 5 °C/min to 250 °C. The column effluent was ionized by electron ionization (EI) at an energy of 70 eV with a transfer temperature of 280 °C and a source temperature of 170 °C. The floral volatiles analysis was performed at the full-bloom stage. The chromatographic running time was 30 min. Three replicates were tested for each variety. The relative quantification of volatiles was calculated using Agilent ChemStation Data Analysis Application based on peak area ratio and the quantity of internal standard. Relative contents (%) = (area under peak/total peak area) × 100.

### 4.3. E-Nose Analysis

A PEN3 portable E-nose (Airsense Company, Schwerin, Germany) was used in the assay. The structure includes a sampling apparatus, a sensor array unit, and pattern-recognition software, as explained previously [54]. The sensor array consists of ten metal oxide semiconductor (MOS) sensors (W1C, W5S, W3C, W6S, W5C, W1S, W1W, W2S, W2W, W3S). The characteristics of the MOS sensors are provided in Supplementary Table S3. The gas in the headspace was thrust over the surface of the sensor for 5 min at a constant flow rate of 150 mL/min. The interval time was 10 s, while the values of the stabilized response sensors were 35–40 s. Three replicates were tested for each variety. For data processing, the stable value of each sensor was extracted.

### 4.4. Identification of VOCs

The floral volatile compounds were identified by comparing them with mass spectra from the NIST Mass Spectral Library (NIST 08), with existing works of literature, and with authentic standards. Mass spectra were obtained by automatic scanning at m/z 20 to 500 amu. The identification of compounds was perceived via comparing the mass spectra with NIST 08 at a match factor of ≥80 [42,51]. The data were processed using mass hunter qualitative analysis workflow software (Agilent Technologies Inc., Santa Clara, CA, USA). Linear retention indices (LRI) of the volatile compounds were measured via an alkane series standard (C7–C40) (Sigma, St. Louis, MO, USA) separated on the DB-5 MS capillary column under the same GC–MS analysis conditions.

### 4.5. Statistical Analysis

Statistical Package for the Social Sciences 19.0 (SPSS Inc., Chicago, IL, USA) was used for the statistical analysis. The differences among samples were calculated via analysis of variance (ANOVA). Data are presented as the mean ± SD (n = 3–5). R 4.0.5 internal statistical functions and the external packages “corrplot” and “mixOmics” were used for the multivariate statistical methods employed (Spearman correlation analysis, hierarchical clustering analysis (HCA), principal component analysis (PCA), and partial least squares (PLS)). Visualization of significant volatile organic compound correlations was performed via a PLS regression network using Cytoscape [61].

## 5. Conclusions

In the current study, we evaluated the floral volatile profile of six *Hedychium* accessions using HS–SPME–GC–MS and E-nose technology. The HS–SPME–GC–MS analysis revealed that (*E*)-β-ocimene, linalool, and methyl benzoate were abundant in highly scented *H. coronarium* ‘ZS’, while eucalyptol was the foremost compound in the medium fragrant *H. forrestii* ‘Gaoling’. Furthermore, analysis of volatile compounds via E-nose technology showed that sensors W1W, W2W, W5S, and W6S played an important role in the recognition of volatile profiles of six *Hedychium* accessions. The correlation analysis between volatile compounds and sensors showed that specific sensors were more sensitive to terpenoids and benzenoids/phenylpropanoids. Our SPME–GC–MS and E-nose will facilitate the researcher in the *Hedychium* breeding program to develop new cultivars of high ornamental traits.

## Figures and Tables

**Table 1:** Volatile compounds detected by HS–SPME–GC–MS in different *Hedychium* accessions.

Name	ID	RT ^1^	LRI Calc ^2^	LRI Nist ^3^	MS ^4^	Relative Content/%					
						ZS	Gaoling	Jin	Caixia	Zhaoxia	KMH
Monoterpenoids	-	-	-	-	-	-	-	-	-	-	-
α-Thujene	M1	8.90	925	923	90	0.09 ± 0.02 b	1.27 ± 0.09 a	0.11 ± 0.02 b	-	0.10 ± 0.03 b	-
α-Pinene	M2	9.05	932	937	91	0.26 ± 0.02 b	3.67 ± 0.74 a	0.23 ± 0.1 b	-	0.08 ± 0.01 b	0.03 ± 0.00 b
Camphene	M3	9.50	949	953	91	0.02 ± 0 b	0.28 ± 0.18 a	-	-	-	-
β-Thujene	M4	10.30	972	966	91	0.83 ± 0.14 b	8.38 ± 2.25 a	-	-	0.48 ± 0.10 b	-
β-Pinene	M5	10.64	977	979	91	-	-	0.99 ± 0.38 a	-	0.24 ± 0.05 b	-
β-Myrcene	M6	10.87	989	990	91	1.45 ± 0.12 b	7.64 ± 1.87 a	0.45 ± 0.16 b	0.38 ± 0.18 b	0.18 ± 0.02 b	-
α-Phellandrene	M7	11.35	1005	1004	80	0.54 ± 0.15 ab	0.86 ± 0.52 a	0.25 ± 0.04 b	0.08 ± 0.03 bc	0.07 ± 0.01 bc	-
α-Terpinene	M8	11.66	1017	1017	96	0.14 ± 0.02 b	0.62 ± 0.03 a	0.07 ± 0.01 c	-	-	-
Limonene	M9	12.26	1029	1028	91	-	-	-	0.08 ± 0.03	-	-
Eucalyptol	M10	12.37	1032	1033	94	8.36 ± 0.48 b	121.03 ± 17.91 a	0.59 ± 0.12 cc	0.12 ± 0.05 d	0.46 ± 0.11 c	-
(*E*)-β-Ocimene	M11	12.87	1047	1040	96	35.58 ± 1.71 a	25.15 ± 5.77 b	16.63 ± 6.25 c	9.62 ± 5.27 cd	2.68 ± 0.62 d	0.04 ± 0.06 e
Cyclopentene, 3-isopropenyl-5,5-dimethyl-	M12	12.98	1057	-	95	0.59 ± 0.26 b	1.67 ± 0.09 a	-	-	-	-
*(Z*)-β-Terpineol	M13	13.56	1071	1145	92	-	0.90 ± 0.24	-	-	-	-
Terpinolene	M14	13.67	1086	1085	93	0.14 ± 0.02 b	0.55 ± 0.12 a	0.19 ± 0.15 b	-	-	-
Linalool	M15	14.35	1099	1102	97	14.98 ± 0.24 a	0.61 ± 0.23 c	4.45 ± 1.32 b	6.98 ± 2.4 b	5.43 ± 0.35 b	0.02 ± 0.04 c
2,4,6-Octatriene, 2,6-dimethyl-, (*E*,*Z*)-	M16	15.04	1131	1131	97	3.33 ± 0.78 a	2.68 ± 0.71 a	1.57 ± 0.29 b	0.47 ± 0.24 c	0.17 ± 0.02 c	-
α-Terpineol	M17	16.78	1196	1199	86	0.16 ± 0.01 b	0.94 ± 0.24 a	-	-	-	-
Dihydro-β-ionone	M18	20.83	1437	1433	98	-	-	-	0.75 ± 0.49 a	-	0.18 ± 0.06 b
Sesquiterpenoids	-	-	-	-	-	-	-	-	-	-	-
α-Cubebene	S1	19.86	1371	1349	96	-	0.05 ± 0.04 a	-	-	0.02 ± 0.01 a	-
Calarene	S2	20.04	1383	1388	87	-	0.3 ± 0.07	-	-	-	-
2-Norpinene	S3	20.40	1438	1436	98	-	0.15 ± 0.08	-	-	-	-
Caryophyllene	S4	20.71	1429	1420	99	0.19 ± 0.08 c	1.2 ± 0.72 bc	1.52 ± 0.52 b	0.49 ± 0.26 bc	2.68 ± 1.2 a	0.45 ± 0.47 bc
(*E*)-β-Famesene	S5	20.93	1454	1456	86	0.06 ± 0.01 b	0.24 ± 0.1 ab	0.14 ± 0.01 b	-	-	1.3 ± 1.12 a
Humulene	S6	21.02	1466	1453	98	-	1.2 ± 0.72 a	-	0.7 ± 0.42 a	0.16 ± 0.07 b	-
Alloaromadendrene	S7	21.13	1470	1461	97	-	0.05 ± 0.04 a	0.05 ± 0.02 a	-	-	-
β-Himachalene	S8	21.30	1471	1500	90	-	0.33 ± 0.29	-	-	-	-
α-Farnesene	S9	21.75	1505	1524	91	1.28 ± 0.24 b	8.2 ± 1.72 a	0.77 ± 0.25 b	0.29 ± 0.05 b	0.65 ± 0.29 b	0.13 ± 0.22 b
α-Amorphene	S10	21.82	1521	1519	94	0.1 ± 0.02 a	-	0.06 ± 0.02 a	-	-	-
δ-Cadinene	S11	21.91	1525	1525	91	0.05 ± 0.04 b	-	0.11 ± 0.02 a	0.09 ± 0.05 ab	-	0.04 ± 0.03 b
Nerolidol	S12	22.33	1563	1562	91	-	0.63 ± 0.5	-	-	-	-
Benzenolds/phenylpropanoids	-	-	-	-	-	-	-	-	-	-	-
Anisole	B1	12.16	1019	1020	83	-	-	0.02 ± 0.02	-	-	-
Methyl benzoate	B2	14.11	1093	1095	95	6.92 ± 3.26 a	-	0.41 ± 0.44 b	0.91 ± 0.67 b	0.2 ± 0.04 c	-
Phenylethyl alcohol	B3	14.75	1112	1110	91	-	-	-	-	0.07 ± 0.04	-
Benzyl nitrile	B4	15.41	1140	1150	93	0.09 ± 0.01 b	-	0.19 ± 0.09 a	-	0.22 ± 0.05 a	-
Eugenol	B5	19.59	1354	1356	95	0.04 ± 0.05 b	-	0.11 ± 0.03 a	-	0.02 ± 0.01 b	-
Phenol, 2-methoxy-4-(1-propenyl)-	B6	20.37	1450	1448	97	0.26 ± 0.24 a	-	0.06 ± 0.03 b	0.12 ± 0.05 ab	0.13 ± 0.08 ab	-
1-Butanol, 3-methyl-, benzoate	B7	20.84	1442	1441	83	0.28 ± 0.03 a	-	0.28 ± 0.18 a	-	0.04 ± 0.01 b	-
Benzyl benzoate	B8	24.33	1780	1760	96	0.03 ± 0.03	-	-	-	-	-
Fatty acid derivatives	-	-	-	-	-	-	-	-	-	-	-
Isobornyl acetate	F1	18.32	1277	-	99	-	1.11 ± 1.27	-	-	-	-
Methyl jasmonate	F2	23.42	1652	1638	92	0.02 ± 0.02 a	-	-	-	0.02 ± 0.01 a	-
Others	-	-	-	-	-	-	-	-	-	-	-
Butyl aldoxime, 3-methyl-, syn-	O1	7.38	850	-	87	1.63 ± 0.43 b	11.1 ± 3.71 a	2.19 ± 1.8 b	0.22 ± 0.14 c	2.73 ± 0.68 b	-
Indole	O2	18.60	1292	1290	81	0.55 ± 0.37	-	-	-	-	-

Note: RT ^1^: Real time. LRI calc ^2^: The calculated linear retention indices. LRI Nist ^3^: Linear retention indices in the literature. Column phase type: DB-5MS. MS ^4^: mass spectrum comparison using NIST libraries. Figures in the table are means and standard error. a, b, c, and d within a row refer to the significant difference (*p* < 0.05). Different letters indicate significant differences among means according to ANOVA analysis (*p* < 0.05). Relative contents (%) = (area under peak/total peak area) × 100; all data are presented as mean ± SD (*n* = 3).

**Table 2:** Response values detected by ten E-nose sensors in different *Hedychium* accessions.

Sensors	Response Values					
	ZS	Gaoling	Jin	Caixia	Zhaoxia	KMH
W1C	0.9802 ± 0.0025 d	0.9668 ± 0.0007 e	0.9887 ± 0.0017 b	0.9835 ± 0.0006 c	0.9885 ± 0.0008 b	0.9928 ± 0.0023 a
W5S	1.4154 ± 0.0100 b	2.0529 ± 0.0498 a	1.2621 ± 0.0176 d	1.4315 ± 0.0137 b	1.3074 ± 0.0062 c	1.1173 ± 0.0049 e
W3C	0.9849 ± 0.0009 d	0.9788 ± 0.0011 e	0.9929 ± 0.0003 b	0.9878 ± 0.0009 c	0.9924 ± 0.0012 b	0.9953 ± 0.0018 a
W6S	1.0082 ± 0.0001 a	1.0024 ± 0.0035 bc	1.0001 ± 0.0014 bc	1.0018 ± 0.0005 bc	1.0018 ± 0.0011 bc	1.0032 ± 0.0013 b
W5C	0.9955 ± 0.0022 a	0.9945 ± 0.0035 a	0.9941 ± 0.0010 a	0.9936 ± 0.0037 a	0.9990 ± 0.0043 a	0.9984 ± 0.0045 a
W1S	1.1539 ± 0.0114 b	1.1864 ± 0.0058 a	1.0777 ± 0.0142 d	1.1193 ± 0.0065 c	1.1054 ± 0.0016 c	1.0645 ± 0.0111 d
W1W	2.9863 ± 0.0414 c	4.7909 ± 0.2573 a	2.4171 ± 0.1133 d	3.6650 ± 0.0539 b	2.8194 ± 0.0465 c	1.6414 ± 0.0091 e
W2S	1.0535 ± 0.0094 b	1.0998 ± 0.0071 a	1.0323 ± 0.0081 c	1.0517 ± 0.0026 b	1.0351 ± 0.0004 c	1.0255 ± 0.0053 c
W2W	2.0548 ± 0.0514 b	2.8989 ± 0.1979 a	1.6443 ± 0.0362 d	2.0564 ± 0.0238 b	1.8660 ± 0.0220 c	1.3316 ± 0.0070 e
W3S	1.0382 ± 0.0009 a	1.0146 ± 0.0151 bc	1.0074 ± 0.0016 c	1.0223 ± 0.0027 b	1.0168 ± 0.0026 bc	1.0212 ± 0.0057 b

Note: The figures in the table are means and standard errors. a, b, c, d and e within a row refer to the significant difference (*p* < 0.05). Different letters indicate significant differences among means according to ANOVA analysis (*p* < 0.05). All data are presented as mean ± SD (n = 3).

## Data Availability

The data is provided in the Supplementary Files.

## Supplementary Materials

The following are available online. Figure S1: Total ion chromatogram of the volatile compounds released from the flowers of six *Hedychium* accessions, Figure S2: A pictorial representation of the number of volatile compounds overlapping in different *Hedychium* accessions, Table S1: The list of floral volatile components in *Hedychium* accessions and their relative contents %, Table S2: The information of six *Hedychium* accessions, Table S3: Sensors used in this study and their main application in PEN3.

## Sample Availability

Samples of the compounds are not available from the authors.

## Abbreviations

E-nose	Electronic nose
GC–MS	Gas chromatography–mass spectrometry
HCA	Hierarchical clustering analysis
MOS	Metal oxide semiconductor
PCA	Principal component analysis
PDMS	Polydimethylsiloxane
PLS	Partial least squares
SPME	Solid-phase microextraction
SSR	Simple sequence repeat
VOCs	Volatile organic compounds
