# Supplementary figures and images for: HS–SPME–GC–MS and Electronic Nose Reveal Differences in the Volatile Profiles of Hedychium Flowers
Source: Molecules. 2021 Sep 6;26(17):5425. doi: 10.3390/molecules26175425 (PMC8433901; doi:10.3390/molecules26175425)

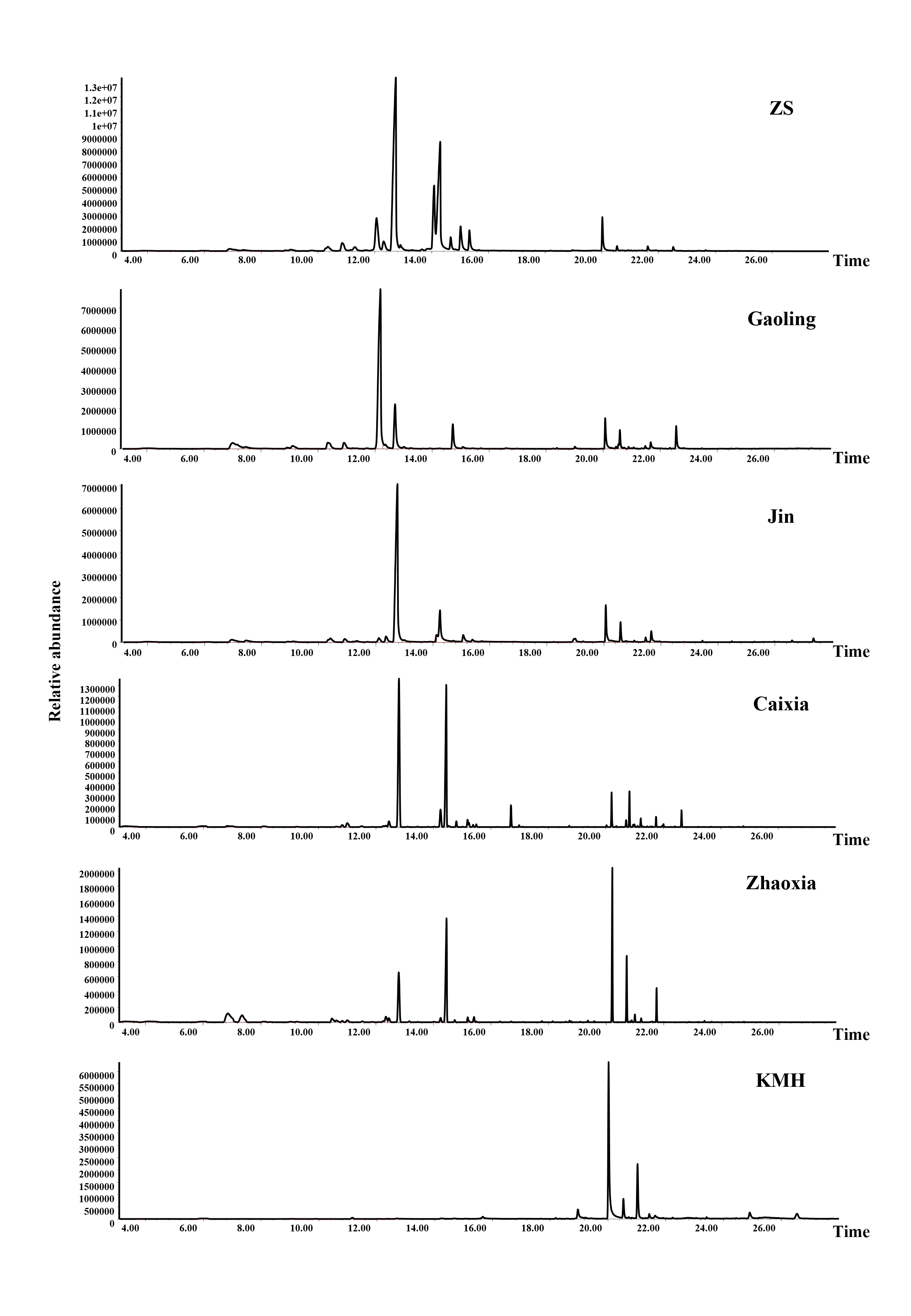

Supplement: Supplementary file 1 [file molecules-26-05425-s001.zip › Figure S1.jpg]

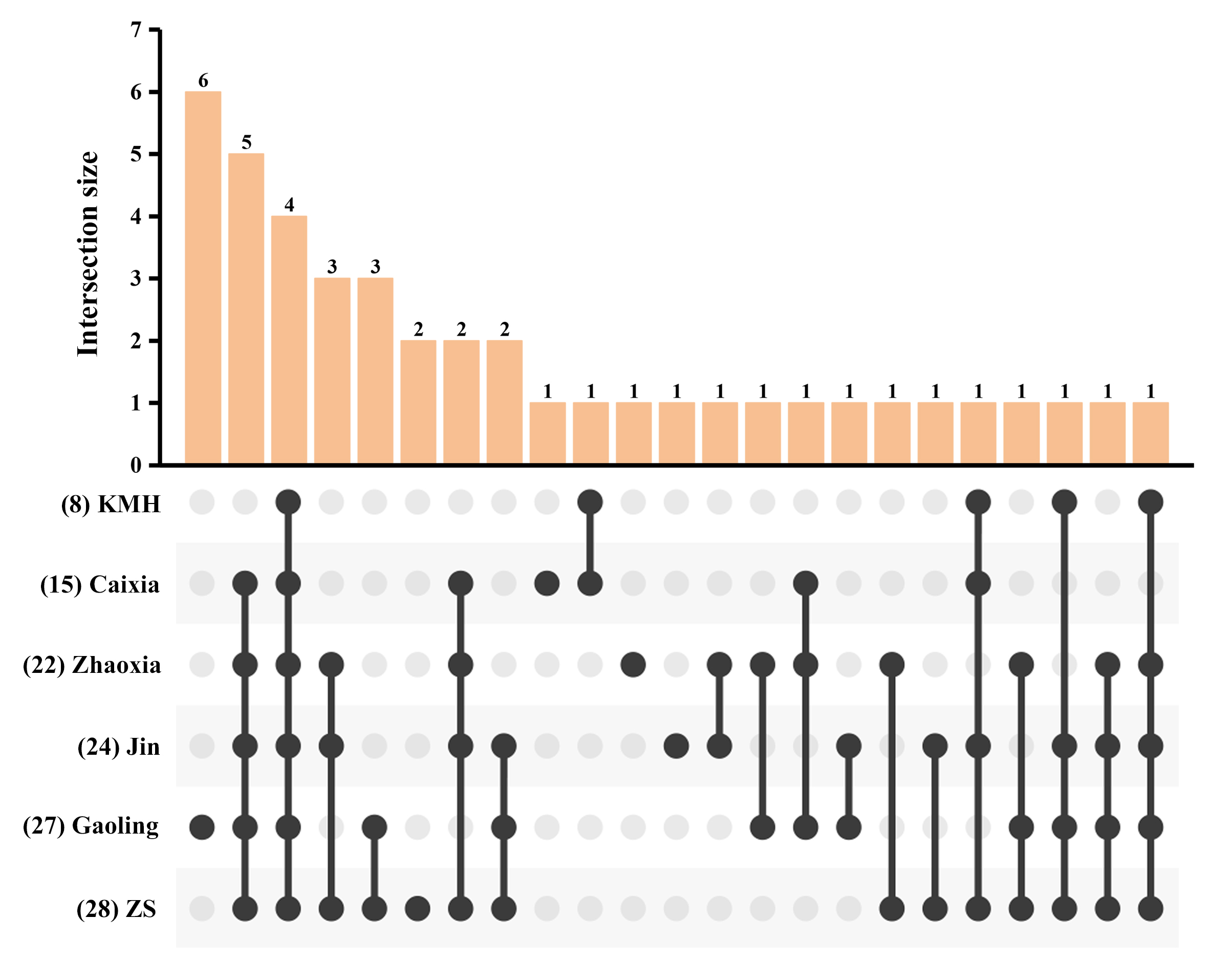

Supplement: Supplementary file 1 [file molecules-26-05425-s001.zip › Figure S2.jpg]
